# The Natural History of Depression in Parkinson's Disease within 30-Month Follow-Up

**DOI:** 10.1155/2015/362892

**Published:** 2015-02-01

**Authors:** Yuan-Yuan Xu, Sheng-Han Kuo, Zheng Liang, Hui Xu, Wu-Ruo Feng, Cui-Yu Yu, Wei-Guo Liu

**Affiliations:** ^1^Department of Neurology, Affiliated Brain Hospital of Nanjing Medical University, Nanjing 210029, China; ^2^Department of Neurology, The Neurological Institute of New York, Columbia University Medical Center, New York, NY 10032, USA

## Abstract

Depression is one of the most common and persistent nonmotor syndromes occurring in 35% of patients diagnosed with PD. However, little information is known about the longitudinal study of its natural history of depression in PD. In this study, we identified 110 patients who are diagnosed with idiopathic PD and recruited them for assessing information about their PD related motor and nonmotor symptoms and rating scales. A follow-up evaluation was performed in 103 patients 30 months later. About 66.7% depressed patients at baseline were still depressed at follow-up, and 24.4% had incident depression among subjects without depression at baseline. Greater decline on MMSE (*P* = 0.029), higher baseline UPDRS-II (*P* < 0.001) score, change of UPDRS-II (*P* = 0.026), and female (*P* < 0.001) were associated with the worsening of HDRS scores. Higher baseline HDRS score (*P* < 0.001) and greater decline on MMSE (*P* = 0.001) were related to the occurrence of depression. In conclusion, cognitive decline is a disease related factor of worsening and the occurrence of depression. Activities of Daily Living (ADL) symptoms in PD and female gender may be crucial factors of increasing depressive symptoms.

## 1. Introduction

Parkinson's disease (PD) affects more than 1% of the elderly people in the world and has been the second most common neurodegenerative disease, just after Alzheimer's disease [1]. Many PD patients, over their course of PD, experience neuropsychiatric disturbances, including depression, anxiety, sleep disturbance, psychosis, and behavior and cognitive changes [2]. Depression is one of the most frequent nonmotor disorders among symptoms of PD patients, occurring in about 35% of PD patients [3]. Depression in PD is considered to be a direct consequence of the neurodegenerative process not merely a reaction to chronic disease [4, 5]. And depressed PD patients show more cognitive impairment and a lower quality of life than nondepressed ones [6, 7]. Despite this, depression in PD is underdiagnosed and undertreated [8]; therefore information about its nature, clinical correlates, and disease course is essential.

Little is known about the longitudinal course of depression in PD. A study [9] that assessed the level of depression in early PD patients every 3 months over 12- to 18-month period, showed that 27.6% of PD patients were screened positive for depression with Geriatric Depression Scale- (GDS-) 15 score and 47% depressed PD patients at baseline experienced remission of depressive symptoms by 6 months. Another study published about 20 years ago [10] reported that 10 of 18 patients with PD (55.6%) who were diagnosed with major depression remained in major depression after 1-year follow-up, six (33.3%) had minor depression at the same point, and only two (11.1%) were in remission from depression. The researchers also identified a positive association between major depression and rapid cognitive decline. Further longitudinal studies are needed to identify the predictors, the course, and prognostic significance of depression in PD patients.

The objective of this study was to evaluate the long-term course of depression in PD patients at 30-month follow-up and to explore the clinical predictors of depression. Since depression and cognitive impairment may be related [10], we hypothesized that cognitive decline would be the predictors of worsening of depression. Motor disorder may also cause negative emotion. Thus our second hypothesis was that higher baseline and increase in motor symptoms and ADL symptoms might relate to depression in PD.

## 2. Methods

### 2.1. Subjects

The study was approved by the Medical Research Ethical Committee of Nanjing Brain Hospital (Nanjing, China). Patients included in this study belonged to a cohort of consecutive patients diagnosed with idiopathic PD who attended the neurology clinic at the Nanjing Brain Hospital from March to September 2009. We recruited 110 PD patients in Hoehr1-Yahr stage 2-3, in this hospital again for interviewing more information about their PD symptoms by using related rating scales, so as to 30 months later. They were diagnosed in accordance with the Brain Bank criteria of the UK PD Society [11] by at least two experienced neurologists. And neurologists with strict training can estimate these scales. All patients had signed the informed consents.

### 2.2. Questionnaires

All evaluations were conducted during a practically defined “on” state. The Unified Parkinson Disease Rating Scale for motor and ADL portions (UPDRS-II and -III) were performed to assess PD related syndromes. The tremor dominant and the postural instability gait dysfunction (PIGD) subtypes of PD were categorized using the criteria described by Jankovic et al. [12]. Mental evaluation was done using the MMSE and HDRS as a screening test for depressive symptoms. Depression was diagnosed by using the Structured Clinical Interview for DSM-IV. And HDRS, as research criteria for depression in PD, is especially developed to assess changes in depression severity based on a clinical interview of the patient and consists of 17 items with a maximum sum score of 53. It was based on a previous study that showed that a cut-off of 13/14 in HDRS is appropriate to detect depression in PD [13]. And MMSE is recommended to be considered as screening tools for dementia in PD [14]. In addition, histories of clinically relevant depression were asked in the interview and the baseline levodopa equivalent dosages were calculated. All assessments were approved by the local ethics committee.

### 2.3. Statistics

Statistical analyses were done with SPSS 19.0 software. Descriptive analyses included means ± SD of normally distributed variables, medians, and IQR of nonnormally distributed continuous data.

Linear regression analyses were conducted to identify predictors of change in depression scores and logistic regression analyses to find predictors of depression at follow-up. We had performed collinearity diagnostic tests before linear regression analyses. Variables associated with depression at the 0.05 level were enrolled in multivariate analyses.

## 3. Results

### 3.1. General Observation between Two Time Points

Of the 110 PD patients, 7 (6.4%) dropped out after the initial evaluation. One of the seven patients died during this period, four patients refused a follow-up evaluation, and two could not be located. The noncompleters were significantly older and experienced longer disease courses than the completers; they also had a significantly older onset age than the completers (Table 1). Ten (9.7%) of the 103 completers used antidepressants at baseline (6 SSRIs, 1 mirtazapine, 2 duloxetine, and 1 subjects received two agents). Three of completers used acetylcholinesterase inhibitors within 30 months. Six subjects had a history of depression before being diagnosed with PD.

The HDRS score showed a statistically significant increase at follow-up (8.66 ± 6.45 versus 11.20 ± 7.62, *t* = −3.363, and *P* = 0.001), and the mean HDRS increment was 2.54 ± 7.68. The proportion with depression at follow-up (33.3%) was significantly higher than at baseline (20.4%) (*χ* ^2^ = 4.192, *P* = 0.041). Among the 21 patients with depression at baseline, 14 (66.7%) had persistent depression, and 7 (33.3%) had remitted depression. Among the 82 subjects without depression at baseline, 62 (75.6%) were still without depression at follow-up, whereas 20 (24.4%) had incident depression. And there were significant differences between depressive proportion at baseline and follow-up (*P* = 0.019) (Table 2).

### 3.2. Linear Regression Analysis

**Figure 1:**
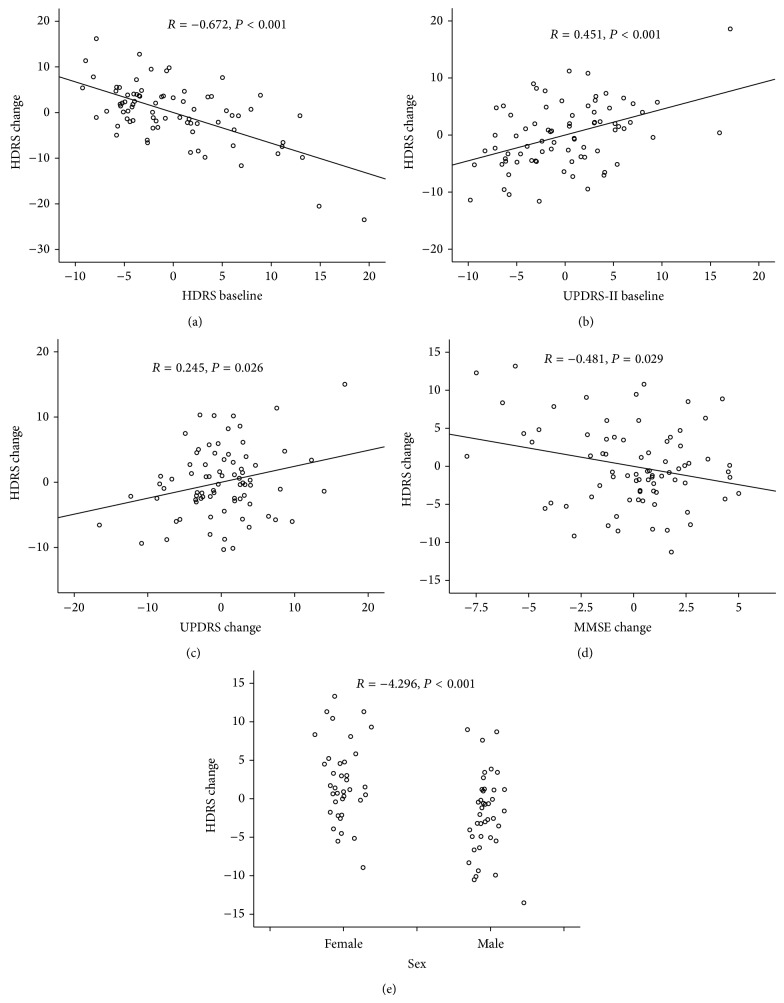
Clinical and demographic correlates of change in HDRS were shown in a linear regression equation. *R* value represents coefficient. Abbreviations: PD: Parkinson's disease; HDRS: Hamilton Depression Rating Scale; MMSE: Mini-Mental State Examination; UPDRS-II: The Unified Parkinson Disease Rating Scale for motor; UPDRS-III: The Unified Parkinson Disease Rating Scale for Activities of Daily Living.

In a multiple linear regression model (Table 3, Figure 1), no associations with change in HDRS score were found for demographic (age, PD duration, PD subtypes, age of onset, history of depression, and living alone), treatment (levodopa dose at baseline, with or without use of antidepressant, acetylcholinesterase inhibitors, or dopamine agonist at baseline), and assessment (baseline MMSE and change of UPDRS-III scale). With only HDRS and UPDRS-II at baseline, UPDRS-II and MMSE changes and sex were found to be significantly related to the change in HDRS. Greater decline on MMSE (*P* = 0.029), more increase in UPDRS-II (*P* = 0.026) and higher baseline UPDRS-II score (*P* < 0.001) were associating with the worsening of HDRS scores. Higher baseline HDRS score was associated with more improvement in HDRS score (*P* < 0.001). And female gender (*P* < 0.001) seems to be more likely to have higher HDRS score.

### 3.3. Logistic Regression Analysis

We have used logistic regression to explore correlates of having depression at follow-up (i.e., HDRS ≥ 14), with the same set of independent variables as mentioned before. With HDRS and UPDRS-II and -III at baseline, changes of MMSE were found significant, but, after adjusting for potential confounders, it showed that only declines on MMSE and HDRS at baseline were significantly correlated with depression in PD. It identified that more rapid decline of MMSE was associated with higher risk for depression. And higher baseline HDRS was also associated with higher risk of having depression at follow-up (Table 4).

To illustrate MMSE decline as a risk factor of having depression in PD, the decline in MMSE in those with incident depression at follow-up was 4.15 ± 3.54, compared to only 1.25 ± 2.76 in those who were without depression symptoms at both assessments (*P* = 0.002). No significant difference has been observed between depression and MMSE score at baseline.

## 4. Discussion

Most studies in different populations have reported the cross-section results about depression in PD. This study assessed the development of clinically significant depressive symptoms in patients diagnosed with idiopathic PD at both baseline and 30-month follow-up.

The prevalence rate of depression in PD at baseline and follow-up is 20.4% and 33.3%, respectively, in line with the review report [3]. One of the main findings in our prospective study was that, of the 103 patients, 13.6% had persistent depression while 60.2% of patients did not experience depression in both assessments. The new incidence of depression was 19.4%, and 6.9% had got into remission after 30 months. These findings are in line with some previous studies. Brown et al. [15] repeated a Beck Depression Inventory assessment 1-year after the first evaluation. The result was that 61.4% showed no depression in both evaluations, while 15.9% were depressed on both assessments, 11.3% were released on the second evaluation, and 11.3% showed the opposite.

There are numerous factors for the emerging and worsening of depression. We found the association between decline in cognition and the aggravation of depressive syndrome and higher likelihood of being depressed at follow-up is of some interest. We also found that, among nondepressed PD patients at baseline, cognition functions were more impaired in depressed patients than in nondepressed patients at follow-up. One explanation might be that cognitive decline and depression may interact between each other. In a research of 245 patients, Tandberg et al. [16] found that cognitive impairment (MMSE < 24) is a risk factor for the occurrence of depression in PD. Other studies have demonstrated that depression can induce and increase cognitive impairment in patients with PD [17]. Depression may cause cognitive abnormalities, and depressive symptoms such as sleep disturbance and social withdrawal can be seen in patients with dementia [16]. Another explanation is that both depression and cognitive impairment might be due to the same neurodegenerative and neurochemical dysfunction [18]. Torack and Morris found that patients with PD, cognitive impairment, and depression had widespread pathological changes in the ventral tegmental area containing the cell bodies of dopaminergic fibers projecting to limbic related cortical and subcortical regions [19]. Also the pathologic substrate for both depression and cognitive abnormalities related to the clinical feature can be summarized as *α*-synuclein pathology in specific neuronal populations anatomically. *α*-Synuclein change in pons (locus ceruleus, raphe, and lateral tegmental nuclei) is related to depression in PD, in basal forebrain (basal nucleus, amygdala, and hippocampus) and the frontal and parietal cortex is related to dementia and comorbid PD [20]. In all, we can only conclude that cognitive decline is one of the risk factors in depression of PD, but we cannot determine whether cognitive changes precede or follow the mood change.

Lower baseline HDRS score was associated with a larger increment in HDRS score. We can explain that lower baseline HDRS score could have a more increase, but most cannot reach the score of 14, while patients with higher HDRS score at baseline most likely remain a score ≥14 despite less obvious increase. We also found higher baseline HDRS score had a higher likelihood of being depressed during the follow-up period which is a significant factor for the occurrence of depression in PD.

Our second hypothesis was that ADL and motor symptoms may relate to depression, since numerous previous studies have similar findings. A study [16] showed a significant correlation of depression with UPDRS subscales I and II but not with the motor subscale. Some scholars [21, 22] also reported the disability in activities of daily living (ADL) maybe more strongly related to depression than the motor disability. Another study has shown an association with predominantly akineto-rigid PD and with bradykinesia [23]. In this study, we found that higher ADL subscale score at baseline and its change might increase depressive symptoms, whereas there were no differences in motor disease severity. In logistic regression, neither ADL nor motor subscale score showed a relationship with the occurrence of depression. Correlations between HDRS and UPDRS that suggest psychological factors secondary to motor impairment might play a role in depressive symptoms in PD. However, while the ADL and motor subscale may not be the only disease related factors of being depressed because some specialists thought that part of depression is a prodromal symptom of PD [24].

We also found that the female appeared to be associated with the increase of depression score, which is consistent with previous reports. Women had a higher frequency of depression, postural instability, and pain, when compared with men [25, 26]. Another study had shown depression to be more common in women in the general population [27]. Such difference could be explained by a different susceptibility of the brain between men and women to depression in PD due to hormonal, genetic, or developmental differences. Distinct social roles that females play may also cause depressive symptoms in PD.

As with most of previous researches [28, 29], we could not find a relationship with depression and advanced age, age at PD onset, PD duration, so as to years of education, PD types, history of depression, and use of dopamine agonist or antidepressant. Only a few patients were taking antidepressants in this study, and influence of such medications on the results may be of small importance. However, some studies have shown that depression in PD is associated with education [22], right but not left onset PD duration [30], predominant akineto-rigid PD, and bradykinesia [23]. These may be due to ethnic and environmental differences.

We acknowledge that our study has a few limitations. First, we only included 12 disease related factors that have the possibility to be associated with PD, thus we may have excluded some relevant factors such as living status and cholineasterase inhibitors. Given the high cost of cholinesterase inhibitors in China, it is not common for PD patients with cognitive decline to be treated. None of the PD patients were treated with donepezil or other cholineasterase inhibitors at baseline and only two patients were treated at follow-up. Because of the small number of patients treated with cholinesterase inhibitors, we did not include this factor in our analysis. And only one patient lived alone and another patient lived in the nursing home at baseline and at follow-up. The rest of patients live with their family, as the common living status of the elderly in China. Therefore, the current proposal cannot address whether living status would be associated with depression in PD. Second, this is a single-center study in Han elderly populations, and the sample size of our cases is small. Therefore, we may have failed to show some significant associations with other factors. Finally, we did not look in between 30 months so some people might develop depression or go into remission within 30 months, so more frequent or longer follow-up is required. The strengths and novelty are that we focus on the natural history of depression in PD and the factors that can be associated with the worsening depressive symptoms, so that we could shed lights in identifying the potentially high-risk patients to develop depression. The attrition rate was low so that we were able to account for more than 90% of the baseline subjects.

Although we have analyzed multiple variables, no straightforward conclusions can be drawn. The complex nature of depression could not be simplified. Biographic factors, family and social support, and life experiences during disease courses should be considered in development of depression in PD. However, these factors are difficult to quantify and interpret.

## 5. Conclusion

This study shows that, in patients with idiopathic PD, depression is common and usually persistent. Thus, depression should be carefully assessed and managed, especially in patients with more cognitive decline who may experience an increased risk of emerging or worsening of depression. Since decline on cognition may also associate with dementia, it is possible that some PD patients with depression and cognitive impairment may end in PD dementia with the progress of the disease course. Decline of daily living ability and female gender are related to increase of depression symptoms. Therefore, our findings await replication by further studies to detect risk factors influencing the incidence and persistence of depression in Parkinson's disease and the treatment of stopping it.

## Figures and Tables

**Table 1:** Baseline clinical and sociodemographic characteristics of completers and noncompleters.

	Completers (103)	Noncompleters (7)	Test value	*P* ^*^
Male (%)	43 (41.7%)	3 (42.86%)	*χ* ^2^ = 0.000	0.994
Mean age, years (SD)	65.83 (9.18)	80.71 (8.20)	*t* = −4.175	0.000
PD duration, years (IQR)	7 (5)	11 (8)	*z* = −2.734	0.006
Onset age, years (SD)	57.69 (10.20)	68.57 (10.06)	*t* = −2.733	0.007
Education, years (IQR)	10.59 (4.04)	9 (3.46)	*t* = 1.015	0.312
HDRS score (IQR)	8 (9)	9 (17)	*z* = −0.853	0.394
MMSE score (IQR)	29 (3)	28 (1)	*z* = −1.836	0.066
UPDRS-II score (SD)	11.03 (6.07)	14.43 (8.28)	*t* = 1.386	0.169
UPDRS-III score (SD)	21.31 (12.16)	26.29 (16.23)	*t* = −1.024	0.308

Values are represented as the mean (SD) of normally distributed variables or medians (IQR) of nonnormally distributed variables. For comparisons of demographics, *P* values were obtained using two-sample *t* tests, *χ* ^2^ test, or rank sum test (*z* value); ^*^ *P* < 0.05 was considered significant. Abbreviations: PD: Parkinson's disease; HDRS: Hamilton Depression Rating Scale; MMSE: Mini-Mental State Examination; UPDRS-II: The Unified Parkinson Disease Rating Scale for motor; UPDRS-III: The Unified Parkinson Disease Rating Scale for Activities of Daily Living portions.

**Table 2:** Patients with or without depression at follow-up.

Baseline	Follow-up	
	Depression	Nondepression
Depression	14	7
Nondepression	20	62

*P* = 0.019; depression group 30 months after versus baseline. *P* values were obtained using *χ* ^2^ test.

**Table 3:** Clinical and demographic correlates of change in HDRS.

Variation	Unstandardized Coefficients	Standardized error	Standardized Coefficients	*t* value	*P* ^*^
Constant	3.669	1.639		2.239	0.028
HDRS baseline	−0.672	0.100	−0.631	−6.736	<0.001
UPDRS-II baseline	0.451	0.113	0.404	3.997	<0.001
UPDRS-II change	0.245	0.108	0.222	2.269	0.026
MMSE change	−0.481	0.216	−0.202	−2.230	0.029
Sex (Female = 0, Male = 1)	−4.296	1.217	−0.307	−3.529	<0.001

We include variations like age, PD duration, PD subtypes, age of onset, and others into multiple linear regression models: using stepwise regression. And model summary: *R* ^2^ = 0.478, *F* = 12.994, and *P* < 0.00. ^*^ *P* < 0.05 was considered significant. Abbreviations: PD: Parkinson's disease; HDRS: Hamilton Depression Rating Scale; MMSE: Mini-Mental State Examination; UPDRS-II: The Unified Parkinson Disease Rating Scale for motor; UPDRS-III: The Unified Parkinson Disease Rating Scale for Activities of Daily Living portions.

**Table 4:** Clinical and demographic correlates of having depression after 30-month follow-up.

	Univariate logistic regression			*P* ^*^	Multivariate logistic regression			*P* ^*^
	*B*	SE	Wald		*B*	SE	Wald	
Sex	−0.683	0.425	2.581	0.108				
Age	−0.017	0.023	0.538	0.463				
Age at PD onset	−0.001	0.021	0.001	0.976				
Education, years	−0.094	0.054	3.059	0.08				
Course, years	−0.108	0.066	2.653	0.103				
PD types (3 types)	0.051	0.225	0.052	0.819				
Levodopa dose at baseline, mg	0.000	0.001	0.096	0.757				
Dopamine agonist (yes/no)	−0.02	0.476	0.002	0.967				
Antidepressant (yes/no)	0.336	0.683	0.243	0.622				
History of depression (yes/no)	−0.947	1.116	0.720	0.396				
MMSE baseline	0.021	0.089	0.055	0.815				
MMSE change	−0.223	0.077	8.338	0.004	−0.357	0.110	10.47	0.001
UPDRS-II baseline	0.093	0.041	5.139	0.023	0.017	0.069	0.064	0.801
UPDRS-II change	0.033	0.038	0.728	0.393				
UPDRS-III baseline	0.040	0.018	4.621	0.032	0.020	0.037	0.298	0.585
UPDRS-III change	0.007	0.013	0.314	0.575				
HDRS baseline	0.109	0.036	9.365	0.002	0.156	0.053	8.753	0.003

First univariate logistic regression was used in every variation, respectively, and then we selected those *P* < 0.05 into a multivariate logistic regression equation. Depression was defined as HDRS ≥ 14 at follow-up. ^*^ *P* < 0.05 was considered significant. Abbreviations: PD: Parkinson's disease; HDRS: Hamilton Depression Rating Scale; MMSE: Mini-Mental State Examination; UPDRS-II: The Unified Parkinson Disease Rating Scale for motor; UPDRS-III: The Unified Parkinson Disease Rating Scale for Activities of Daily Living portions.
